# Natural Computing Applied to the Underground System: A Synergistic Approach for Smart Cities

**DOI:** 10.3390/s18124094

**Published:** 2018-11-22

**Authors:** Clemencio Morales Lucas, Luis Fernando de Mingo López, Nuria Gómez Blas

**Affiliations:** ETSI Sistemas Informáticos, Universidad Politécnica de Madrid, Madrid 28031, Spain; clemencio.morales.lucas@alumnos.upm.es (C.M.L.); nuria.gomez.blas@upm.es (N.G.B.)

**Keywords:** urban public transport system, smart city, natural computing, collective computation

## Abstract

The management and proper use of the Urban Public Transport Systems (UPTS) constitutes a critical field that has not been investigated in accordance to its relevance and urgent idiosyncrasy within the Smart Cities realm. Swarm Intelligence is a very promising paradigm to deal with such complex and dynamic systems. It presents robust, scalable, and self-organized behavior to deal with dynamic and fast changing systems. The intelligence of cities can be modelled as a swarm of digital telecommunication networks (the nerves), ubiquitously embedded intelligence, sensors and tags, and software. In this paper, a new approach based on the use of the Natural Computing paradigm and Collective Computation is shown, more concretely taking advantage of an Ant Colony Optimization algorithm variation and Fireworks algorithms to build a system that makes the complete control of the UPTS a tangible reality.

## 1. Introduction

**Figure 1:**
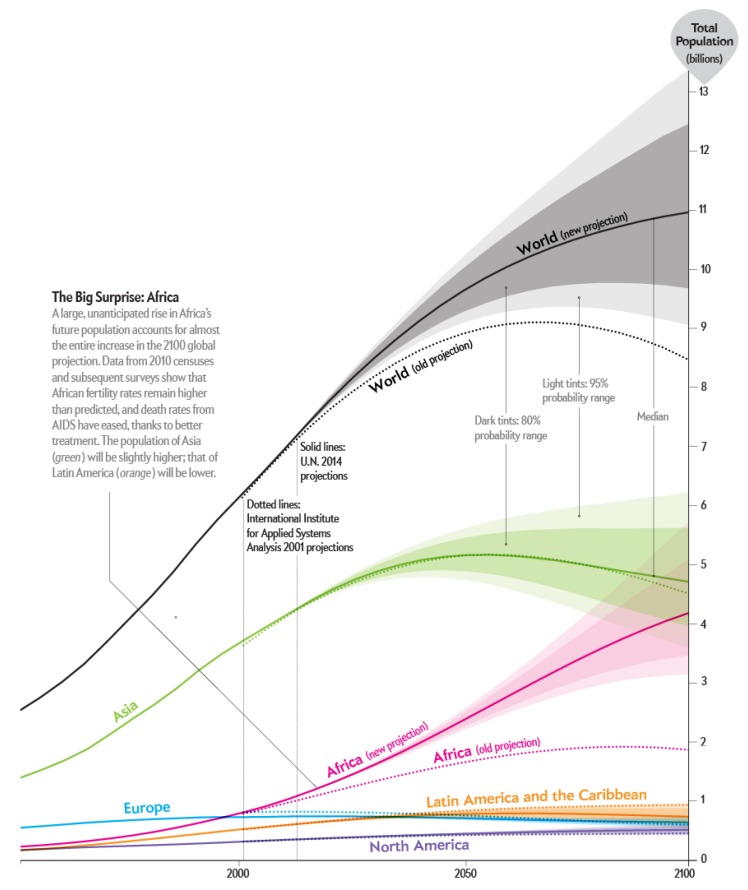
World Population estimated growth between the years 1950 and 2100. These charts show estimates and probabilistic projections of the total population for countries or areas, geographical aggregates and World Bank income groups as defined in Definition of Regions. The population projections are based on the probabilistic projections of total fertility and life expectancy at birth, based on estimates of the 2017 Revision of the World Population Prospects. These probabilistic projections of total fertility and life expectancy at birth were carried out with a Bayesian Hierarchical Model. The figures display the probabilistic median, and the 80 and 95 per cent prediction intervals of the probabilistic population projections, as well as the (deterministic) high and low variant (+/−0.5 child) of the 2017 Revision of the World Population Prospects [1,2].

Since their pioneering conception in 1829, underground trains have changed in in so many ways. From the 47 km/h that Stephenson’s train reached in the aforementioned year, to the 310 km/h that the Spanish AVE is capable of obtaining, trains have experienced an evident impact regarding their technology. However, these changes have not been applied to the management system and conception of the underground itself as it is nowadays. On the one hand, the rapidly growing massification of the world’s urban cores together with the intensive use by citizens of the underground, is pushing the transition of these cores to the Smart City purest concept, where every single element within the city has ratiocination enough for it to be called intelligent. In the year 2050, 66% of the world’s population is expected to be living in urban cores (United Nations, Department of Economic and Social Affairs, New York, NY, USA), increasing the current percentage of 54% by 12%. In other words, the current estimations show that the continuous urbanization process that the world is facing, along with the overall growth of the world’s population, will add another 2.5 billion people to urban populations by 2050, with close to 90% of the increase concentrated in Africa and Asia, according to a new United Nations report. To sum up, 66% of a world population of 9 Billion (5.94 Billion) will be living in urban cores in 2050. The aforementioned massification can be seen in Figure 1.

On the other hand, it is important to note that this need has been outlined by organisms such as C.E.O.E (Spanish Confederation of Business Organizations, Madrid, Spain). In fact, as described in CEOE [3]:

This frame of sustainability and efficiency that must involve the Smart Cities, has a direct relationship with other key areas, such as […] the efficient management of the mobility of people […] [Cities are lacking] Indicators for the collection appropriate measures […] [Cities systems need] real-time knowledge about incidents, and an improved efficiency and management of the public transport.

It is, therefore, evident that cities nowadays need a deep improvement on their IT systems and infrastructure, evolving to new schemes where data is seen as a binder for the city. To contribute to this goal, a gathering and management system based on Natural Computing is presented on this paper.

Even the concept of Smart City is still being under constant redefinition, most authors agree that many different individuals, agents, and devices, operate with their environment within the Smart City realm [4,5,6,7]. Therefore, as R. G. Hollands [8] points out, the relation among all these elements will define the behavior of the Smart City itself. It is easy to realize that an important area of the Smart City will be based on the interaction between its different components with their environment. This fact disembogues in a Socio-Collective Interaction, where the Smart City in general terms, and specially the underground system beneath, can be seen as a huge swarm, where agents collaborate with each other [9]. The aforementioned approach justifies the present investigation project, based on a change in the way of tackling the management processes of any underground system, using Collective Computation algorithms [10,11,12] instead of the classical, graph-oriented ones [13].

## 2. Definition of the Problem

**Figure 2:**
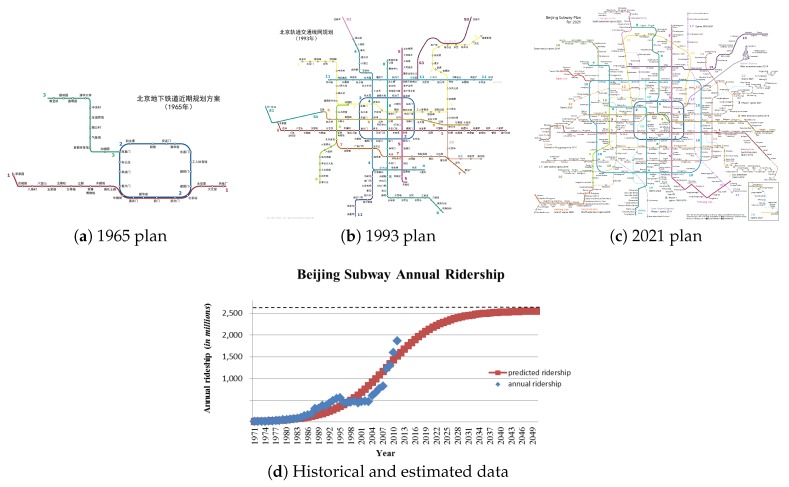
Beijing Subway estimated growth between the years 1970 and 2021. (**a**) Schematic map of Beijing Subway lines in 1965. (**b**). Lines in operation by 1995. (**c**) A map showing Beijing subway lines in operation by the end of 2017 (solid lines) and subway lines then projected for completion before the end of 2021 (dashed lines). (**d**) Historical and estimated data.

The underground system beneath any urban core is a living, constantly changing entity. According to the Annual Subway Ridership of the Metropolitan Transportation Authority (Metropolitan 2016), 3.410 billion itineraries were made last year within Beijing’s underground system, more than the double of New York city’s subway itineraries of 1.763 billion. This number is massive but it is expected to increase drastically in years to come. If we study the number of passengers using Beijing’s subway, the 1800 million passengers/year it has nowadays, is expected to increase by 700 million by the year 2050. In other words, 27% of the world population will be using Beijing Subway by 2050. The aforementioned increase in Beijing’s subway usage is shown in Figure 2.

Thus, how can these itineraries be traced, letting the management know who is using the underground and when? How can the users rapidly know if there is an emergency or a path, which is not working due to technical errors within the underground? How can we analyze the massive data that can be potentially generated by so many itineraries? The response to these questions is precisely the reason that justifies the investigation, which aims to create a synergy of elements achieved by the application of many innovative Computation Paradigms ([14,15]). These paradigms, in conjunction with strict software control, which will operate with user’s smartphones, will create to an increase in the intelligence of the Urban Public Transport Systems (UPTS hereinafter).

Regarding the law-related aspects, it is important to remark that, according to the Regulation 2016/679 of the European Parliament and of the Council of 27 April 2016, dissociated data (that information related to a physical person that does not allow for his direct identification) can be used in these sort of systems [16]. As it is frequently seen and widely accepted in social networks, users will accept the share of their dissociated data by using the application for statistical, non-commercial purposes. This acceptance is shown in examples such as Facebook and WhatsApp, that hold 1871 and 1000 million users, respectively [17]. However, they will have the right to reject this safe data sharing. Rights of Access, Rectification, Cancellation and Opposition regarding dissociated data, as well as any kind of data stored, will be granted at any given time as in Spanish Organic Law 15/1999 [18] which is aligned with European Union (EU) laws. Moreover, Data Suppression, Limitation and Portability will be always granted as well, as data storage good practices suggest in EU.

## 3. Investigation Goals

One of the most important aspects in a Smart City is the optimal use of available resources. Sensors can help to make an optimal use of resources by taking thoughtful decisions after analyzing their environment. These sensors can control, detect, and manage the unnecessary use and make certain adjustments as needed.

At present, the major cities waste up to 50% of water due to pipe leakages. With sensors fitted on each pipe, water leaks can be easily detected and corrected before any heavy loss. Besides this fact, the irrigation systems in public parks can automatically turn off whenever rain is detected to save water. Sensors have also enabled the concept of “Advanced Metering Infrastructure (AMI)” underpinning energy management in cities. Cities are considering the use of “Smart Meters” embedded with Phase Measurement Unit (PMU) sensors and a communication module which facilitates a two-way communication between the consumer and the supplier. For utility service providers, it helps check meter status prior to sending a repair crew in response to a customer call. These checks prevent the needless dispatch of field crews to customer sites. For consumers, it can provide the real-time energy usage details in a way that can be easily understood. Based upon this data, users can change the preferences and make more informed decisions about their usage without waiting for their energy bill at the end of month. In cities, street lights remain on even when there is no activity in the area (sometimes in daylight too). Additionally, it becomes very difficult for authorities to detect any fault and theft of street lights. With sensors, lights can go dim when they are not needed, and authorities can get a text message instantly whenever there is a fault or tampering in street lights. With sensors fitted in the garbage bins, the municipal authorities can be notified when they are close to being full. The Netherlands became the first ever to produce “Intelligent Bins” that report to the officials via text messages, whenever the bins are either full or if there is any damage. Traffic can be reduced with sensors that detect where the nearest available parking lot is. Motorists get timely information via text messages so they can locate a free parking slot quickly, saving time and fuel. A similar project is being carried out in San Francisco called SFPark, where parking spaces have been installed in 8200 on-street places. This concept would be replicated in several other states in coming days. Sensors mounted on poles can monitor the Ambient Air Quality (AAQ) of cities. Citizens can monitor the pollution concentration in each street of the city or they can get automatic alarms when pollution beyond a certain level.

The present investigation has the objective of fixing, chiefly the following goals that define an accurate overview of the investigation:

- Investigate the Computing Paradigms according to the realm of the Collective Collaboration: As it will be explained in further sections within this document, the Natural Computation stands as the best ally when it comes to this investigation aspect [19]. Genetic Algorithms [20,21], Ant Colony Optimization [22], Swarm Computing [23], Grammatical Evolution [24] and Grammatical Swarm [25], which have been widely investigated in order to find improvements, if any, in parallel investigations about algorithms and optimization.
- Find a nexus between the Computing Paradigms involved and the problem to solve: Once a strong theoretical overview has been given to the reader, the union point and nexus with the chased system will be described. Please bear in mind that, at the time being, there is no application of these algorithms in the UPTS context, factor that increases the innovative character of the present investigation.
- Design and development of a system that, using the needed paradigms within Natural Computation, allows a wide study of the behavior of the underground users: In a nutshell, the system aims to become a tool that makes possible the study of the user’s behavior, by taking dissociated data up in order to guard the privacy of the citizens. This objective will be possible thanks to the UPTS users’ Smart phones, for which an application is to be developed in the Android Operating System.
- Make precise studies about the statistical population that uses the UPTS. The dissociated data provided by commuters is a constantly updated and accurate source of information about the most frequent profiles in the UPTS. This information can be used as feedback for the system itself, establishing the exact number of people using it.
- Know, in an accurate way, the most popular routes for the users, as well as their behavior between the UPTS. It is important to note that this factor constitutes an open door for an efficient management within the system. It is important to note that the term efficient differs to effective in a subtle, but crucial manner; while an effective system achieves every objective, an efficient one achieves every objective as well but, in the best, optimal way. The study of the most popular routes can be seen as a first step to increase the frequencies of the highly crowded routes as needed.
- Prepare, thanks to the estimations gathered from the statistical study of the data, the UPTS to deal with peaks. Such scenario can be predicted by attending at atypical values within the data set gathered by the system.
- Detect anomalous situations, such as a blocked train within a tunnel, or different scenarios where the number of users standing at the platform is high enough to fear an accident, surpassing the capacity of the specific dock.
- Prepare alternative routes in case of intensive use and/or fault of the UPTS systems. The system can detect whether a route is too crowded or not, allowing the UPTS to prepare alternative routes, if possible. The same solution applies in case of system fault.
- One of the main virtues of the system lies on it high level of customization.

## 4. Overview of the System

Smart Cities are environment friendly as they use sustainable materials for building facilities and reduce energy consumption. Efficient use of technology helps create an efficient transport management system, improve healthcare facilities, and develop a robust communication network to connect all businesses, people and beyond the relationships between central and national levels of governments. A network of sensors, cameras, wireless devices, data centers form the key infrastructure that allows civic authorities to provide essential services in a faster and more efficient manner.

There will be an urban environment that permanently communicates with citizens and is capable of managing public services in real time to improve their quality of life through traffic management, garbage collection, waste disposal, irrigation systems, assisted parking, and that alerts the local authority when an incident occurs so that the government can stay in touch with the people.

The platform is divided into a layered architecture associated with the different functional blocks. The following section describes the components and main features of the architecture:

**Layer 0**. Integrate sensing/performance technologies. The platform will facilitate integration sensing technologies currently available, regardless of their nature, and will provide an open environment that allows the dynamic addition of new sensor systems and technologies. To accomplish this, the platform will provide data encapsulation mechanisms that standardize the information received from sources such as Wi-Fi, ZigBee, etc. Layer 0 of the platform is a broker that defines communication with sensor networks of different natures (Wi-Fi, ZigBee, Bluetooth, etc.), and obtains the raw data from sensor networks. The main novelty of this layer is the ability to provide the platform and the upper layers with openness regarding the connection to sensor networks of different natures, and thus ensure that upper layers of the architecture have access to information and can perform data fusion at different levels.

**Layer 1**. Low-level services. Given the information exchanged with the environment through layer 0 as described above, the existing functional requirements and a set of low-level services will now be defined; specifically, those that depend on the types of networks and technologies integrated into every deployment. After obtaining the raw data, a gateway is provided, defined through adapters that allow the information received to be standardized. In this first stage, the platform provides services such as filtering of signals, normalization services or other treatment services at the basic level signals. These services are provided by the adapters and will be associated with algorithms that perform initial treatment of the data, so that it can be presented to higher layers in a more homogenized way.

**Figure 3:**
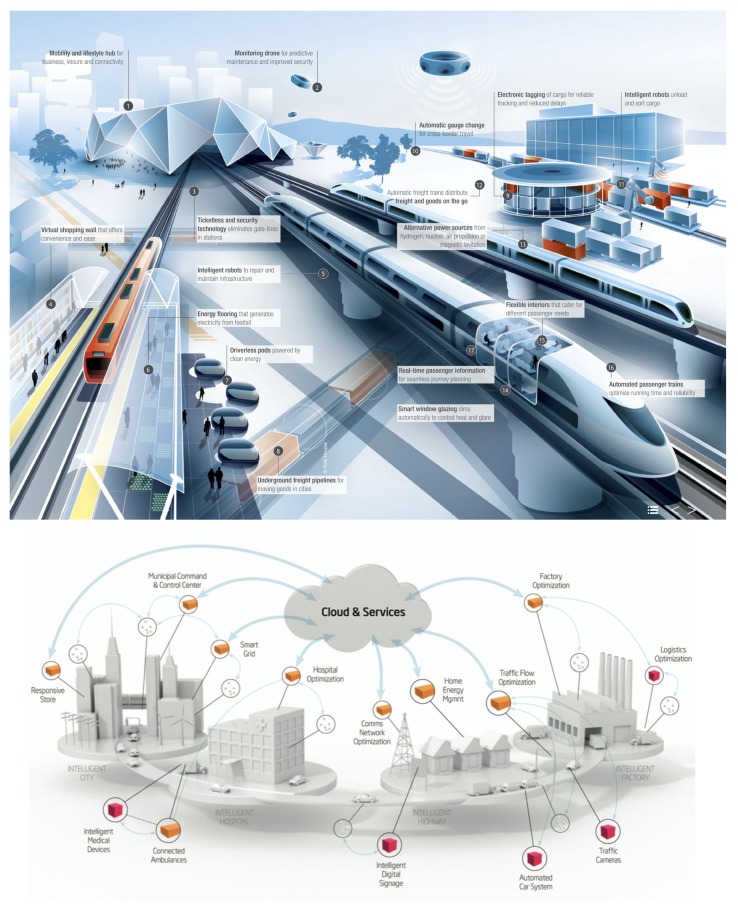
Different views of Smart Cities. Currently in-development system, including fixed smart dust, mobile smart dust, users’ smart phones, control center and system administrator could be easily integrated. (Reprinted with kind permission from http://www.cnet.se/smart-cities and https://www.arup.com/projects/rail).

**Layer 2**. Information fusion algorithms. Each organization includes the roles required to facilitate an intelligent management of the information obtained from the lower levels of the architecture. The MAS incorporates agents specifically designed to interact with low-level services. In addition, we propose the design of intelligent swarm agents specialized in Information Fusion (IF) using ant colony systems, Fireworks algorithms, and Particle Swarm Optimization. The system to be developed formed by five groups of elements, has the main structure that can be seen in Figure 3. There are five groups of elements within the system, which are described below:

- *A*: Fixed Smart dust: In wireless sensor networks terms, this smart dust element, that will be unique in each UPTS station, will behave as the sink node. This special device will be integrated in the dock itself, receiving information from its Bn counterparts. The sink node will be the only element able to establish communication with the Operations and Control Center, *D*, and the elements within the *C* set.
- B[1,2,3,⋯,n]: Mobile Smart dust: The present element of the system, embedded in the UPTS trains fleet, will behave as a slave of the sink smart dust. They will establish communication with the sink and will receive data from the elements within the *C* set, which will be explained below.
- C[1,2,3,⋯,n]: Users smart phones: This fundamental element within the system will be used by the users to make evident their presence in the dock. The elements within this group will be able to communicate with the sink smart dust, *A*, as well as with its counterparts in *B*, the mobile smart dust devices, that will make possible to know the number of users in the train. Please note that the set formed especially by *A* and B[1,2,3,⋯,n] will shape the wireless sensor network of the system, that will operate closely with the C[1,2,3,⋯,n] devices. A mobile application will be developed for the elements in *C*, that will retrieve the dissociated data of the users, let them know different routes in case of massive congestion, configure itineraries and show warning regarding abnormal situations that may occur within the UPTS.
- *D*: Operations and Control Center (OCC): This element will behave as the management point within the system realm, receiving the data sent by the smart dust in every single station, showing the pertinent status and the presence, if appropriate, of abnormal situations from the safety/systems failure point of view.
- *E*: System Administrator: Evaluator of the data showed by the OCC. Will operate accordingly to the UPTS current status and its environment, whether triggering a specific security protocol against failure or solving the different spurious scenarios that may occur.

### Sensors Technology

Simulations using sensor-collected data would assist planners in identifying potential traffic challenges at granular levels–street, intersection, freeway ramp, and other locations. Congestion problems could be addressed more efficiently, cutting down the number of planning errors in the process. High-tech methods leveraging the Internet of Things can aid in cutting down traffic congestion. For example, existing technologies can detect smartphone Bluetooth signals (short range) and Wi-Fi signals (longer range) from vehicles as they pass through points where sensors detect and record their presence. By placing sensor detectors at key locations along roads, transportation managers can determine the general path of vehicles as they pass through these points. Having this greater insight into traffic flow and congestion points could help city planners to identify opportunities to smooth traffic flows and plan the infrastructure more accurately to support a city’s growing needs.

Most commuters have smartphones and many vehicles today have built-in electronics (Wi-Fi, Bluetooth, ZigBee) emitting signals that can be captured. Scanners in the streets, typically attached to street lights, can capture these signals. The number required will vary with the amount of traffic flowing through an area, distances between vehicles, and the level of reflection created by nearby signs, billboards, or any obstructions. These scanners need to be positioned in a pattern that increases the chance of detection. Once the data is captured, the raw information contained in Bluetooth, Wi-Fi, and ZigBee data logs can be aggregated to provide greater insights into traffic patterns. Swarm intelligence holds a great potential to totally transform the way traffic patterns affect our daily lives and our daily commutes. It is now up to city planners throughout our major metropolises to recognize the benefits of these simulations and to create the necessary infrastructure.

## 5. Algorithms Involved

Despite its apparent disparity, the following Computation Paradigms and the Algorithmic Techniques described below fall within the spectrum of the Natural Computation Paradigm. As long as the investigation is currently on a medium stage, the nexus with the system of some of these paradigms, as well as their application to the system, are still being under investigation.

As the accustomed reader will surely intuit, the algorithmic entities attached to this paradigm have, as their main base, the logic associated with phenomena present in nature, as well as the logic associated with the genetic-molecular base of the living beings, thus. As it can be read in Handbook of Natural Computing [26], we can formally define Natural Computing as the set of computing techniques that circumscribe to, at least, one characteristic defined within the following group:

- Obtain its base from observing nature, establishing a computing simile.
- Base its reasoning in the use of the computers to synthesize natural events.
- Use natural materials, from the logical or physical point of view, such as DNA strings or chromosomes, to achieve its computational processes.

### 5.1. Genetic Algorithms

**Figure 4:**
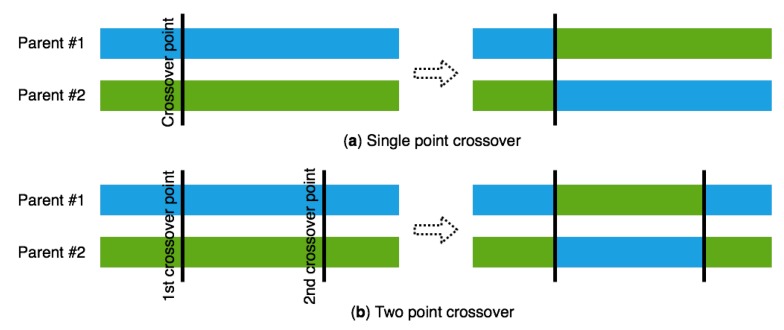
Genetic operator crossover between alleles. (**a**) Single point crossover in which one crossover point is selected, binary string from beginning of chromosome to the crossover point is copied from one parent, the rest is copied from the second parent. (**b**) Two-point crossover in which two crossover point are selected, binary string from beginning of chromosome to the first crossover point is copied from one parent, the part from the first to the second crossover point is copied from the second parent and the rest is copied from the first parent.

As stated by Charles Darwin in his opus magnum On the Origin of the Species [27], from immemorial times living beings have been forced to a continuous evolutive process looking for survival. Every single specie evolves from a common ancestor looking for the adaptation to its environment and survive, following the process named natural selection. In a parallel way, Genetic Algorithms (GA hereinafter) follows the same pattern, trying to evolve a population. Thus, as it can be extracted from John H. Holland’s Adaptation in Natural and Artificial Systems [20,21], a GA can be formally defined as a set of ordered instructions, that aim to achieve a specific problem, which are based on the genetic-molecular base of the evolutive process of the living beings. It is remarkable that, despite the paternity of the GA is attributed to Prof. Holland, his sublime work means the colophon to the investigation cycle started by the distinguished Gregor Mendel, with his laws stated in Experiments in Plant Hybridization [28], based on the investigation over Pisum Sativum. In his publication, Mendel describes, using this specific pea variation, the basic rules related to the characteristic’s transmission between individuals through genetic inheritance. Actually, a GA has the objective of evolving certain specimens that set a population. To chase this goal, the GA uses random operations that establish a simile with the natural processes related to biological evolution. These methods, called genetic operators, are the following:

- Selection: In this operator, the GA chooses individual genomes from the population to start a later breeding process. Selection can be made by means of various techniques, as seen in A Comparison of Selection Schemes Used in Evolutionary Algorithms [29]. These techniques can be Roulette-Wheel Selection, Selection by Truncation, Selection by Ranking or Selection by Tournament, to quote a few of them.
- Crossover: Process whereby a variation in the chromosomes is done from a generation towards the following one. It is remarkable that, following the natural simile, the crossover mocks the sexual reproduction of the living beings. Letting a binary string be the information to be represented, there are several crossover techniques, and they all produce permutations in the chromosome. Seeing the chromosome as a set of alleles, the technique of crossover in a point can be an illustrative example; as shown in the following figure, once a bit within the chromosome is selected, every successive allele is exchanged between a chromosome and its pair, generating a new offspring in the process, see Figure 4.
- Mutation: Variation within the genotype of a living being. Represents the action of the mutagens present in the ecosystem. It is remarkable that the genetic unit able to mutate is the gene, atomic, inheritable unity of data that builds up an individual’s DNA.
- Recombination: Process whereby a DNA portion is cleaved to provide its further union to a different genetic material molecule. It is important to note that this action provokes different genetic permutations in a specie regarding its predecessors, producing chimeric alleles. This advantage makes the sexual reproduction possible between living beings, while avoiding Muller’s ratchet (Named after its discoverer, Hermann Joseph Muller, is the process by which the different genomes of an asexual population accumulate deleterious mutations in an irreversible manner, that may result in the irrevocable extinction of the specie).

### 5.2. Ant Colony Optimization

**Figure 5:**
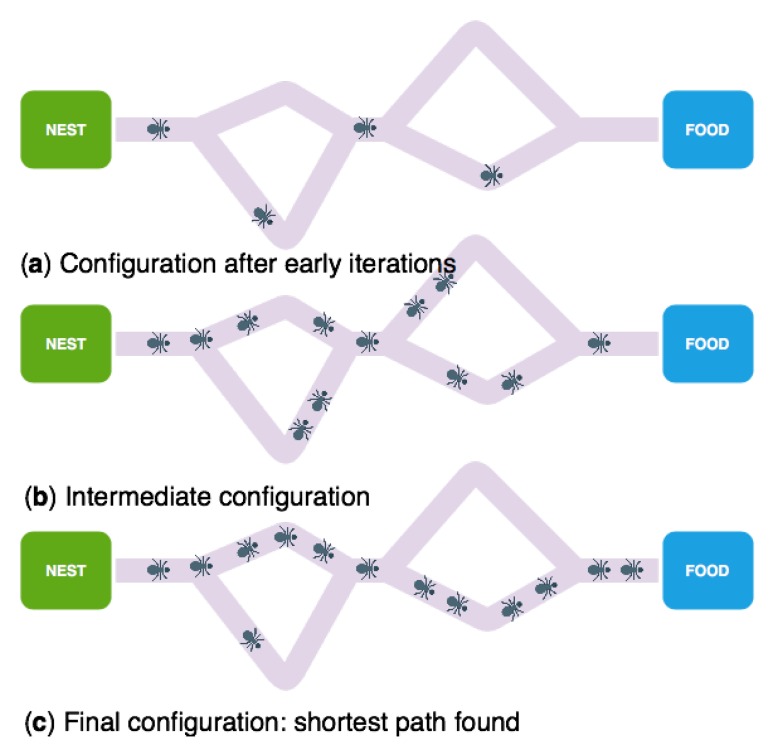
Goss Experiment representation (double bridge experiment). A colony of ants selecting the best route between the nest and food, (**a**) Schematic of the bridge and ants in early stages, (**b**,**c**) Final configuration where shortest path is found, note some “miss” ants due to the stochastic behavior of the colony.

As Marco Dorigo and Gianni Di Caro establishes several times along [30], Ant Colony Optimization (ACO hereinafter) is the name that refers to a multi-agent paradigm where every agent’s behavior is inspired on the ant idiosyncrasy when searching for livelihood. The algorithms that fall within this classification are based on the Goss Experiment (see Figure 5), using an Iridomyrmex humilis colony. In this experiment, the ant nest is connected to a livelihood source by means of two different paths, where one is longer than its counterpart, as the following figure shows:

After allowing the ants to freely move themselves along the scenario, it can be seen that, after an initial moment, they always choose the optimal, shorter path to the livelihood source. It is remarkable that, as well, this experiment demonstrates that a route selection probability is directly proportional to the length difference between both paths.

After studying the results thrown by Goss Experiment, a question arises; How do all the ants know what is the shortest path? The answer to this question is based on the concept known as stigmergy. The aforementioned concept alludes to those collaboration protocols, through the physical medium, where the different components collaborate due to the accumulation of objects or magnitudes in the environment, such pheromones or humidity. This concept is, precisely, the main tool within ant’s communication; as the ants go backward and forward to the livelihood source, they deposit a chemical substance called pheromone. As it happens in several species, this substance provokes specific reactions and behavior in the individual counterparts, allowing to know what the shortest path is.

It is remarkable that the directive that makes each ant *k*, placed in the *i*-th node, using a pheromone trail $\tau_{ij}$ in order to calculate the probability it has to use to choose a node *j* that belongs to *N*, as well as the following node where it has to move along, where $N_{i}$ constitutes the set of nodes adjacent to *i*, is given by the equation:

(1) $$p_{ij}^{k}=\left\{\begin{array}{cc} \tau_{ij} & j\in N_{i}\\ 0 & j\notin N_{i} \end{array}\right.$$

### 5.3. Particle Swarm Optimization

Since the dawn of science, many scientists have been intrigued by a movement, as elegant as optimal, present in nature: The harmonious synchrony in bird flocks and fish shoals, where the individuals are able to move without even rub with each other, despite the hundreds, thousands of elements in certain cases, of individuals present in these sets. Thanks to scientific investigation, it has been demonstrated that, apart from this optimal movement, these animals present certain swarm patterns in their behavior.

Concretely, it is important to highlight the hyperbolic interest of Grenander Heppner on their opus magnum A stochastic nonlinear model for coordinated bird flocks (Grenander, Di Caro 1999), where both zoologists synthesize their investigation referred to the nature-hidden directives that mark the asynchronous movement of the bird flocks, changing its direction suddenly in the presence of predators and tacitly regrouping, among other interesting abilities. In the same line, Reynolds Flocks, herds, and schools: a distributed behavioral model [31] stands out, aiming to the study of the interesting choreography that birds deploy.

Clustering the aforementioned references as base, the Particle Swarm Optimization (PSO, hereinafter) paradigm is known as the technique that pretends to optimize a problem due to a meta-heuristic strategy, which is, due to the iterative trial of improving a candidate solution with regards to a pre-stipulated quality criterion. Thus, in a way that reminds to GA, PSO optimizes a problem starting from a set of candidate solutions, typically particles over the space, moving them along through the searching space without forgetting the premises of PSO mathematical base, which involves the position and the speed of the particles. As can be inferred, the technique mimetizes the group behavior of the aforementioned living beings, where each individual movement is influenced by the best local position known, while, in a parallel way, the swarm maintains a best global position known. This best global position is updated by the best position known by all the individuals in the swarm, fact that will guide the set to move searching for the best global position.

PSO adopts a tiny number of postulations along its execution process, exploring a large search space. Despite from that, PSO is a meta-heuristic, so it is not possible to adamantly ensure that the algorithm is going to find an optimal solution of the problem for every single case. In a more mathematical, accurate way, PSO does not use the gradient of the tackled problem, which means that this technique does not require the problem to be differentiable, as well as it happens in typical optimization methodologies such quasi-Newtonian methods or Gradient Descent. Thus, PSO can be used, enjoying a high success rate, in optimization problems that are especially non-regular, where there is certain ambient noise, or those presenting a dynamic, changing-over-time behavior. PSO algorithm pseudocode can be stated as follows:

Let the following symbols represent properties of a particle:

- $x_{i}$ is the current position of particle *i*
- $v_{i}$ is the current velocity of particle *i*
- $p_{Best}$ is the personal best position of the particle
- $g_{Best}$ is the global best particle
- $c_{1}\in R$ is the personal influence (acceleration coefficient)
- $c_{2}\in R$ is the global influence (acceleration coefficient)
- $r_{1}$ and $r_{2}$ are random numbers distributed using a uniform pattern on interval [0,1].

With these notations, the formula to calculate a particle’s velocity at time t+1 (time is simulated using iteration number) is:

(2) $$v_{i}\left(t+1\right)=v_{i}\left(t\right)+c_{1}*r_{1}*\left(p_{Best}-x_{i}\right)+c_{2}*r_{2}*\left(g_{Best}-x_{i}\right)$$

where, $r_{1}$ and $r_{2}$ are randomly generated for every velocity update and $0\leq r_{1},r_{2}\leq 1$. They should both be different each iteration. In addition, $c_{1},c_{2}$ are user defined values called acceleration coefficients where $0\leq c_{1},c_{2}\leq 2$. Their value depends on the problem to be optimized.

**Figure 6:**
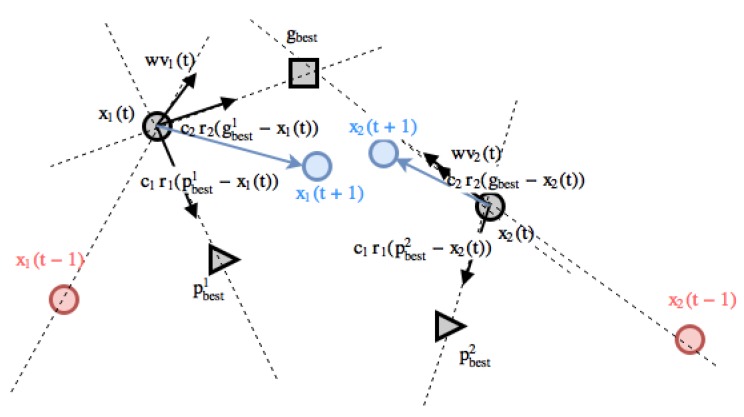
Particle swarm optimization model: position update using a star topology ($g_{best}$) and an inertia *w* term. Please note that $g_{best}$, $p_{best}^{1}$ and $p_{best}^{2}$ are fixed in time *t* in order to make the figure as simple as possible, their position could change (or not) depending on the fitness value of particles $x^{1}$ and $x^{2}$ along time in a real example.

New particle position at time t+1 just adds the newly calculated velocity to its current position at time *t*. In other words, the position now is the previous one adding its velocity, see Figure 6. Note that $g_{Best}$ refers to a star topology.

(3) $$\begin{array}{c} x_{i}\left(t+1\right)=x_{i}\left(t\right)+v_{i}\left(t+1\right) \end{array}$$

Algorithm 1 shows the standard PSO process to update location and velocity of particles.

**Algorithm 1:** Standard particle swarm optimization algorithm: update process. **for** Each time step *t*
**do**  **for** Each particle *i* in the swarm **do**   update position $x_{i}\left(t+1\right)$ using Equations (2) and (3)   calculate particle fitness $f(x_{i}\left(t+1\right))$   update $p_{Best}$ and $g_{Best}$  **end for** **end for**

### 5.4. Fireworks Algorithm

As history shows, mathematical optimization has always been a field under investigation (Boyd, 2004). More concretely, and due to its vital importance on Computer Science, the search processes involved in computing problems have been widely investigated. These processes are highly related to sorting algorithms too.

A search algorithm is a set of mathematical instructions aiming to place a custom order between the elements within a collection. Optimization techniques, as well as efficient sorting, has been the main objective when it comes to these kinds of algorithms since the first investigations on the field [32].

In a similar way, Fireworks Algorithm (FWA onwards) belongs to the Swarm Intelligence spectrum and takes its inspiration by observing fireworks explosion [33,34]. This algorithm is proposed for the optimization of complex functions, and its implemented following an accurate simulation of fireworks explosion process. FWA takes special attention to keep the diversity of sparks as it will be explained, by maintaining two different search processes. In general, terms, FWA constitutes an approach to explore a massive search space. This search is based on the search of random points confined by a certain distance measure that hopes one or more of the points of interest will yield promising results. Once these points give interesting results in terms of the mathematical function to be optimized, a more concentrated search will be spawned in the near points, iterating through the algorithm until an optimal solution is found.

Regarding FWA operating mode, the algorithm starts with the selection of a concrete number, *N*, of initial locations. *N* fireworks will be thrown at these locations, and the fireworks will consequently throw sparks. The location of these sparks will be retrieved to evaluate its quality, and in case the optimal solution has been found, the algorithm will finish. Otherwise, FWA will set another *N* fireworks at *N* locations, spawning and iterating repeatedly over the aforementioned process until the optimal solution is found.

**Figure 7:**
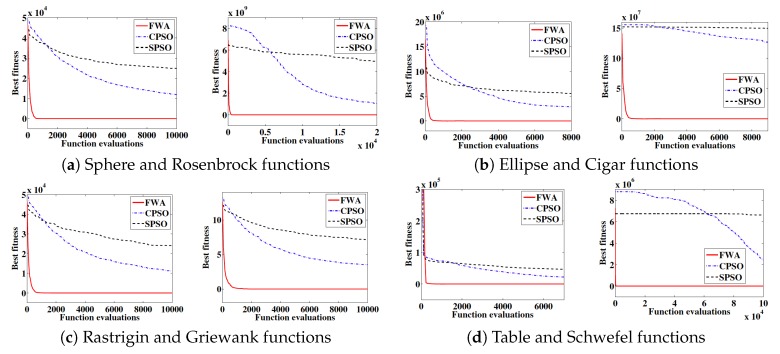
Convergence curves of the FA, the CPSO and the SPSO on eight benchmark functions. The function fitness is averaged over 20 independent runs. Reprinted from [35], with kind permission from Springer Science + Business Media. Copyright © Springer-Verlag Berlin Heidelberg 2010.

To validate the convergence curves of FWA, Clonal Particle Swarm Optimization (CPSO) and Standard Particle Swarm Optimization (SPSO) are taken [33]. A set of eight different benchmark functions (Sphere, Rosenbrock, Ellipse, Cigar, Rastrigin, Griewank, Tablet and Schwefel) averaged over 20 independent runs are thrown on these algorithms, reaching the conclusion that FWA presents a higher speed compared to CPSO and SPSO, see Figure 7.

The information shown in the last figure, with the addition of Ackley benchmark function, can be analyzed in the following figure, where the Statistical Mean and Standard Deviation for FWA are far more agile compared to CPSO and SPSO, see Table 1.

To sum up, FWA finds brilliant solutions with 1000 times of function evaluations, reflecting the quick convergence speed of the algorithm against CPSO and SPSO. This algorithm is therefore a strong ally when it comes to Smart Cities environment applications in a computing-related way, being currently investigated to create a system capable of searching lost people in the Smart City rural surroundings.

FWA has multiple applications, as [34] shows with a system for data mining low-rank applications such web search. These systems reduce storage and requirements at runtime, as well as the noise regarding data representation when it comes to essential associations. The Non-negative Matrix Factorization, whose parameters are widely explained at (NMF, [36]) arrives to a low-rank approximation that verifies that non-negativity constraints are satisfied. NMF approximates a data matrix by where and are the NMF factors. NMF requires all entries in, and to be zero or positive. The following figure shows the FWA-based optimization algorithm pseudo code for NMF—see Algorithm 2-, showing explicitly the high level of configurability associated with FWA, where SIO stands for Swarm Intelligence Optimization.

**Algorithm 2:** Pseudo code for the initialization procedure for NMF factors *W* and *H*. The two for-loops in lines 4 and 10 can be executed concurrently [33]. Given matrix $A\in R^{m\times n}$ $H_{0}=rand\left(k,n\right)$ % Compute in parallel **for**
i=1 to *m*
**do**  Use SIO to find $w_{i}^{r}$ that minimizes $\left|\right|a_{i}^{r}-w_{i}^{r}H_{0}{\left|\right|}_{F}$, (${min||.||}_{F}$ of row *i* of *D*) **end for** % Gather $W=[w_{1}^{r},\cdots ,w_{m}^{r}]$ % Compute in parallel **for**
j=1 to *m*
**do**  Use SIO to find $h_{j}^{c}$ that minimizes $\left|\right|a_{j}^{c}-Wh_{j}^{c}{\left|\right|}_{F}$, (${min||.||}_{F}$ of col *j* of *D*) **end for** % Gather $H=[h_{1}^{c},\cdots ,h_{n}^{c}]$

## 6. Application to the Smart Cities Realm and Potential Results

It is inferred from this paper that the wide spectrum of applications that can be extracted from Natural Computing and applied to the Smart City is massive, see [37,38]. Moreover, as it has been pointed out along the paper, the cities need deep changes to be called ”smart”. If in short terms Natural Computing mimics phenomena present in nature, it turns out to be an excellent ally when improving the different systems that belong to a city: A city can be seen as a swarm of individuals that operate among a system. Thus, following a bottom-up scheme, when it comes to a city’s realm, the citizens can be seen as particles, therefore they conform a swarm, atomic work element of the Natural Computing paradigm. This simile opens the Smart City concept to be widely improved by applying Natural Computing, where Genetic Algorithms, ACO, PSO and Fireworks Algorithm especially stand out. It is important to point, though, that this paper has motivated parallel investigation lines in the authors, and thus another way of thinking when it comes to the particle-people simile is being under investigation. In this parallel approach, people that share the same information is being grouped in sets of particles, spawning the concept of a more complex type of ’super-particles’ among the swarm [39].

On the one hand, the efforts under the current investigation are being currently driven into the ACO Algorithm spectrum: Even efficient, ant pheromone is simple, primitive; it only marks the shortest path to the livelihood source, but; what if this pheromone concept is extended to a super-pheromone? A super-pheromone will store dissociated data of a person (i.e., age, gender, education level, etc.), thus, it will be possible to know which person profile is transiting for each UPTS section by seeing the user as an ant. More concretely, by applying the schema shown in Figure 3. Overview of the currently under development system, under the Section 4, the UPTS will improve its perception, knowing who is circulating where, and consequently showing publicity screens according the relevant information for the public. (For instance, it will be more effective to show the publicity related to a new video-game near an institute area when the train is crowded by young people, while a new credit card with certain bonuses will be more appropriate in the UPTS section beneath the financial area of the city.)

On the other hand, GA paradigm is being used to evolve a route instead of a chromosome population: by means of a Smartphone application, users will be able to quickly know the best route between two points in the UPTS, as well as backup routes in case of systems breakdown. PSO can be used for studying the data retrieved in the OCC. This will make possible to optimize the system by applying a statistical investigation over the data, detecting statistical outliers, and acting in consequence. It is remarkable that the investigation regarding this slope and other Natural Computing paradigms is in an early stage, thus new applications are susceptible to emerge.

### 6.1. Wireless Sensor Networks

Wireless Sensor Networks consists of spatially distributed autonomous sensors to monitor physical or environmental conditions, such as temperature, sound, vibration, pressure, etc., and to cooperatively pass their data through the network. Researchers of wireless sensor networks face challenges such as communication failures, memory/computational constraints, and limited energy. Many issues in wireless sensor networks could be formulated as multidimensional optimization problems, and approached through bio-inspired techniques.

- PSO is a simple, effective, and computationally efficient optimization algorithm. It could be applied to address WSN issues [40] such as optimal deployment, node localization, etc.
- ACO is based on ants that are mobile agents that migrate from one node to an adjacent one searching for feasible paths between source and destination nodes [41]. Ant colony solution components correspond to network nodes, and, accordingly, routing tables correspond to pheromone tables in which each pheromone variable holds the estimated goodness of selecting a neighbor to forward a data packet [42,43].

#### Routing, Energy and Deployment Using Ant Colony Optimization

Like all routing algorithms, ant colony algorithms for mobile ad hoc networks, the information related to routing is organized into the so-called routing tables. These tables contain the used information by the algorithm in its forwarding of local decisions. The kind of information contained, as well as the way in which it is used and updated depend solely on the characteristics of the algorithm. The routing table is in turn a local database and a local model of the global state of the network [43].

A local routing, instead of storing the whole network graph, will be more suitable to keep track of the information going to a destination node. The local routing table in every node/mote keeps the following information:

- A list of neighborhood nodes/motes that have Internet connection or act a sink node, see Table 2. This table is build using a discovery ant that every node will run, when the ant reaches the Internet sink a backward ant will be sent back to the source node that updates the probability and lookup table of nodes. These discovery ants could start at regular time intervals or when a network configuration change happens.
- To be able to send back data packets, the MAC address or ID of the source node must be kept in the path of the route to the Internet sink. That is, every node/mote stores the pair:
  - the MAC/ID of the source of every transmitted packet (to be able to send data back to the source),
  - and the MAC/ID of the connected node/mote of transmitted packet (to send data back).
  Data packets are sent using the Internet lookup table, according to the probability of the node. When the echo information passes a node and reaches the source, then the probabilities are also updated.

The probability of Internet lookup table at node *i* that has a radio link with node *j* is updated using the following equation:

(4) $$p_{ij}=\frac{\tau_{j}\alpha_{ij}\beta_{j}}{\sum_{j}\tau_{j}\alpha_{ij}\beta_{j}},$$

where $\tau_{j}$ is the pheromone information updated by backward ants, $\alpha_{ij}$ is the radio link power between nodes and $\beta_{j}$ is the node power status.

This approach does not consider low power consumption [44,45] and does not consider memory limitations, this can be solved using a circular table to remove low probabilities. Real-time information is not need and backward information could also use a circular table when there are a lot of nodes.

Energy of optimization path in WSN using ACO could be compared to energy of gradient-based routing [46,47]. Based on simulation results the energy-aware routing protocol with optimization scheme shows better performance than gradient-based routing protocol with optimization scheme [42]. It offered an efficient multi-path data broadcast to achieve reliable transmission in the case of node error. It has been managed to enhance the life time of the wireless sensor network by efficiently broadcasting the data. This implies that an ACO could be efficiently used to solve the network routing problem with reduction in energy consumption to maximize the lifetime of the wireless sensor network. Energy routing protocol with ACO gives best performance as compared to gradient-based routing protocol using ACO.

Wireless sensor networks deployment problem refers to determining positions for sensor nodes such that the desired coverage, connectivity, and energy efficiency can be done with as few nodes as possible [47]. Events in an area devoid of an adequate number of sensor nodes remain unnoticed; and the areas having dense sensor populations suffer from congestions and delays. Optimally deployed WSN assures adequate quality of service, long network life and energy saving. Available PSO solutions to the deployment problem are computed centrally on a base station for determining positions of sensors, mobile nodes, or base stations.

Scale and density of deployment, environmental uncertainties and constraints in energy, memory, bandwidth, and computing resources pose serious challenges to the developers of WSNs. Issues of node deployment, localization, energy-aware clustering, and data-aggregation are often formulated as optimization problems. Most analytical methods suffer from slow or lack of convergence to the final solutions. This calls for fast optimization algorithms that produce quality solutions using less resources. PSO has been a popular technique used to solve optimization problems in WSNs due to its simplicity, high quality of solution, fast convergence, and insignificant computational burden. However, iterative nature of PSO can prohibit its use for high-speed real-time applications, especially if optimization needs to be carried out frequently. PSO requires large amounts of memory, which may limit its implementation to resource-rich base stations.

## 7. Conclusions and Discussion

In this paper, a new scheme for endowing intelligence to a city UPTS is given, chasing the transition of the city to a Smart City. In this approach, Natural Computing paradigm will be applied to the system, after a deep investigation that aims to improve the involved paradigms, if possible. Despite the investigation still being in an early stage, the system is likely to improve the data gathering related to the UPTS, allowing the pertinent authorities to improve the system and even monetize the information gathered by the system under development. Moreover, users will be able to enjoy a better use of UPTS, knowing alternative routes in case of systems breakdown and being able to travel in an efficient way. Swarm intelligence holds a great potential to totally transform the way traffic patterns affect our daily lives and our daily commutes. It is now up to city planners throughout our major metropolises to recognize the benefits of these simulations and to create the necessary infrastructure.

## Figures and Tables

**Table 1:** Statistical mean and standard deviation of solutions found by the FA, the CPSO and the SPSO on nine benchmark functions over 20 independent runs of 10000 function evaluations [34]. Copyright © IGI Global 1988-2018.

Function	FA’s Mean	CPSO’s Mean	SPSO’s Mean
	(StD)	(StD)	(StD)
Sphere	0.000000	11,857.425781	24,919.099609
	(0.000000)	(3305.973067)	(3383.241523)
Rosenbrock	19.38330	2,750,997,504.000000	5,571,942,400.000000
	(11.94373)	(1,741,747,548.420642)	(960,421,617.568024)
Rastrigin	0.000000	10,940.148438	24,013.001953
	(0.000000)	(3663.484331)	(4246.961530)
Griewank	0.000000	3.457273	7.125976
	(0.000000)	(0.911027)	(0.965788)
Ellipse	0.000000	2,493,945.500000	5,305,106.500000
	(0.000000)	(1,199,024.648305)	(1,117,954.409340)
Cigar	0.000000	122,527,168.000000	149,600,864.000000
	(0.000000)	(28,596,381.089661)	(13,093,322.778560)
Tablet	0.000000	15,595.107422	42,547.488281
	(0.000000)	(8086.792234)	(8232.221882)
Schwefel	4.353733	8,775,860.000000	6,743,699.000000
	(1.479332)	(1,217,609.288290)	(597,770.084232)
Ackley	0.000000	15.907665	18.423347
	(0.000000)	(1.196082)	(0.503372)

**Table 2:** Sample of a local routing table store in a random sensor. Please note that (Mote ID:Probability) is the routing forward data and (Source Mote, Route) is the routing backward information.

Internet Lookup Table	Data Packet Information
(Mote ID: Probability)	(Source Mote, Route)
7:0.9	(2,3)
5:0.6	(2,5)
3:0.3	(2,1)
1:0.4	(2,6)
